# Genomic Context and Mechanisms of the *ACVR1* Mutation in Fibrodysplasia Ossificans Progressiva

**DOI:** 10.3390/biomedicines9020154

**Published:** 2021-02-05

**Authors:** Roberto Ravazzolo, Renata Bocciardi

**Affiliations:** 1 Department of Neurosciences, Rehabilitation, Ophthalmology, Genetics, Maternal and Child Sciences (DiNOGMI), University of Genova, 16100 Genova, Italy; bocciardi@unige.it; 2 UOC Genetica Medica, IRCCS Giannina Gaslini, 16100 Genova, Italy

**Keywords:** Fibrodysplasia Ossificans Progressiva, ACVR1, human genome, *cis*-regulatory elements, genetic modifiers, gene expression

## Abstract

Basic research in Fibrodysplasia Ossificans Progressiva (FOP) was carried out in the various fields involved in the disease pathophysiology and was important for designing therapeutic approaches, some of which were already developed as ongoing or planned clinical trials. Genetic research was fundamental in identifying the FOP causative mutation, and the astonishing progress in technologies for genomic analysis, coupled to related computational methods, now make possible further research in this field. We present here a review of molecular and cellular factors which could explain why a single mutation, the R206H in the *ACVR1* gene, is absolutely prevalent in FOP patients. We also address the mechanisms by which FOP expressivity could be modulated by *cis*-acting variants in the *ACVR1* genomic region in human chromosome 2q. Finally, we also discuss the general issue of genetic modifiers in FOP.

## 1. Introduction

Research in rare diseases is of paramount importance to build knowledge for the design of potential therapeutic approaches. For Fibrodysplasia Ossificans Progressiva (FOP), the state of the art of research is highly consistent with such relationships between research findings and their application to clinics in term of therapeutic strategies. The International Fibrodysplasia Ossificans Progressiva Association (IFOPA, https://www.ifopa.org/ (accessed on 4 February 2021)) indicates a list of 16 therapeutic proposals, among them some already developed as ongoing or planned clinical trials (https://clinicaltrials.gov (accessed on 4 February 2021)). All of them derive from research on FOP pathophysiology and, because the cause of the disease is a mutation in the *ACVR1* gene, encoding the Alk2 receptor for Bone Morphogenetic Proteins (BMPs), which induces an anomalous activation of signaling, the majority of the proposed therapeutic strategies are focused on the inhibition of BMP signaling.

Most of the research directed to find ways to inhibit the BMP signaling took advantage of the availability of murine models. A very recent article published in the Special Issue “Fibrodysplasia Ossificans Progressiva: Studies on Disease Mechanism towards Novel Therapeutic Approaches” of Biomedicines [1] describes in detail several murine models utilized in studies on FOP. Besides mice, another animal model, zebrafish, has provided important information about the first inhibitor of BMP signaling [2] and, very recently, about the relationship between the *ACVR1* mutation and its ligand dependency [3]. FOP models were also generated in Drosophila [4]. Many cellular models have been utilized to investigate FOP disease mechanisms, which were useful for studying many peculiar aspects of FOP pathophysiology but had the inevitable limitations intrinsic to the model itself. However, a fundamental advancement in cell biology techniques, the induced Pluripotent Stem Cell (iPSC) derived from FOP patients, proved to be suitable for application in the study of the disease and was used to demonstrate the role of Activin A in BMP signaling starting from the mutated ACVR1 receptor [5], also reported in an article that described the first conditional *ACVR1(R206H)* murine model [6]. The FOP iPSCs were subsequently used for further characterization of mechanisms and drug screening [7,8].

Important efforts have been devoted to research on pluripotent cell types which can be sensitive to inducing signals and differentiate into bone [9,10,11,12], and we think that future studies in this field will provide additional information on the contribution of different cell types in the FOP osteogenic process.

Among the determinants of FOP onset and progression, other topics in the FOP pathophysiology are important to understand the disease mechanisms and possibly become target of potential innovative therapeutic approaches. The local environment in which osteogenesis ultimately takes place is a complex assembly of multiple cell types and molecular components which play different roles in the FOP pathogenesis and, because of the complexity, much needs to be investigated and clarified. Local inflammation is present in all sites where new bone will be formed, sometimes with clear apparent signs in typical flare-ups, but also in an underhand manner, which is now possible to perceive thanks to modern diagnostic techniques like MRI coupled to PET-CT [13]. Pathological examination of early FOP lesions showed the presence of cells of innate immunity, lymphocytes, macrophages, and mast cells (reviewed in Shore, 2010) [14], and the large array of inflammatory molecules released in the local environment are described [15]. We think that many features of local inflammation in the FOP lesions have to be further investigated to clarify their role in the disease pathogenesis and, therefore, become potential targets of therapeutic intervention. Hypoxia associated with inflammation in the FOP lesion is one of the factors which has been described as an important contributor to heterotopic ossification (HO) [16] with potential application for therapeutic approaches. The fibroproliferative process described in the evolution of the FOP local lesion [14] is responsible for extracellular matrix production and tissue stiffness, which appears to be increased in FOP compared to the repair of muscle damage taking place in the absence of ACVR1 mutation [17,18] and to be another factor favoring HO in the local FOP lesion. We remark that all different cell types which play a role in the local environment where the HO develops in FOP are carrying the *ACVR1* mutation, and that such mutation is probably influencing to some extent their function, increasing the complexity of pathogenic factors that intervene in the onset and progression of the disease.

FOP, like many other genetic disorders caused by single gene mutation, shows variability in clinical manifestation, like age of onset, frequency and outcome of flareups, localization of heterotopic bone and others, which can depend on several reasons, among them genetic/genomic factors. In the recent past, DNA sequencing was a reliable procedure to find gene mutations causative of such diseases (see Shore, 2006 for FOP) [19]. Nowadays, genome-wide sequencing studies can be carried out with advanced technology and lower cost, which can allow us to investigate the genome searching for genetic factors that influence disease penetrance and expressivity.

In this article, we present a speculative analysis of two aspects of the FOP pathogenesis related to the *ACVR1* mutation itself: (a) why a single mutation has such a high prevalence in FOP patients; and (b) how the expression of *ACVR1* can be modulated by *cis*-acting variants in the surrounding genomic region of the gene in human chromosome 2q, thus affecting FOP expressivity. We also discuss the general issue of genetic modifiers in FOP.

## 2. The *ACVR1* Mutation in FOP

FOP is caused by mutations in the *ACVR1* gene, encoding the Alk2 Bone Morphogenetic Protein Type I receptor. More than 95% of affected individuals carry a heterozygous recurrent mutation, a G to A substitution at nucleotide c.617 that changes a CGC codon encoding arginine to CAC, encoding histidine (R206H) [19]. This residue is located in the GS domain of the receptor protein. The remaining small percentage of patients carry different mutations in the same GS domain or in the kinase domain of the protein [20,21,22].

The very high recurrence rate of the c.617G>A mutation stimulates interest in understanding the reason of such recurrence. In most cases the mutation is a de novo event likely occurring in a germ cell, since very few cases are described as inherited from an affected parent with an autosomal dominant pattern [19]. A rare case of germ cell mosaicism is also described in two half sisters having the same mother and different fathers [23]; mosaicism caused by a *de novo* mutation in a post-zygotic phase cannot be excluded, although no evidence of such an event is available in the literature.

**Figure 1:**
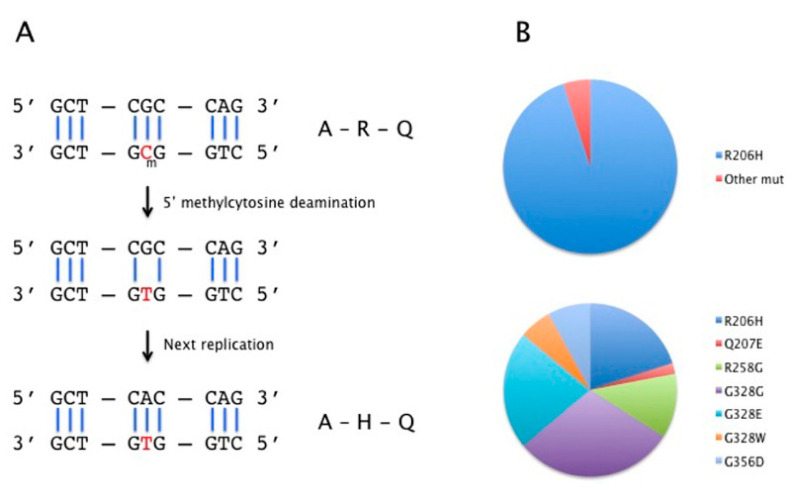
(**A**) Mechanism of the G>A mutagenesis; (**B**) Percentage of ACVR1 mutations in Fibrodysplasia Ossificans Progressiva (FOP) (upper) and in Diffuse Intrinsic Pontine Glioma (DIPG) (lower). The red color in part A indicates the interested nucleotide.

To explore the reasons for the high recurrence rate of the c.617G>A substitution, we have to consider the context in which this type of mutation occurs and its effect on the cell where it takes place. The base substitution occurs in a CpG dinucleotide in a coding exon of the gene devoid of CpG high density (CpG island). This base change is the most common among nucleotide substitutions [24], due to the high mutation rate at CpG dinucleotide where cytosine is frequently methylated (5-mC). 5-mC then undergoes spontaneous deamination that results in a C>T change and a G:T mismatch that, in the following replication step, is fixed as a G>A transition in the interested codon (Figure 1). Interestingly, this mutation was not exclusively found in humans. The same identical mutation in *ACVR1* was reported in two cats with FOP [25], confirming the general validity of this mutational mechanism.

Besides FOP, mutations in the *ACVR1* gene have been described as somatic events in cancer tissues from 20–30% of patients affected with Diffuse Intrinsic Pontine Glioma (DIPG) [22,26,27]. It is interesting to observe that ACVR1 in DIPG is mutated in the same gene domains as in FOP but, unlike FOP, the very high frequency of c.617G>A is not detected in those somatic mutations (Figure 1), implying that the cell and the time when the mutational event occurs make a difference. The fundamental point is that the highly recurrent FOP mutation occurs in germ cells where mutation is a very rare event compared to somatic cells; the DNA methylation level is subjected to variation in the different phases of germ cell development and the processes of male and female germline development vary. It is generally stated that the majority of germline de novo mutations have a paternal origin and that an increasing paternal age favors de novo mutations in the offspring (see Goldman, 2019, as a comprehensive review) [28], due to the continuous division of spermatogonial cells generating replication errors and failure to repair that is progressive with age. An article published in 1979, long before the discovery of the genetic cause of FOP, reported a paternal age effect [29]. Compared to spermatogenesis, oogenesis is concluded before birth and no further cell divisions take place after birth. However, maternal-derived germline de novo mutations also occur, particularly in some genomic regions [30,31], which would imply that, in those cases, the mechanism of mutation is different from replication errors. It is also reported that CpG sites in cells of the male germline are methylated at a higher rate than in the female germline, making the sites more prone to spontaneous deamination and C>T transitions [32].

In highly recurrent de novo mutations, like the c.617G>A causative of FOP, named “selfish” mutations, it has been proposed that they confer spermatogonial selection leading to clonal expansion of mutated spermatogonia. Examples of such mutations are found in *HRAS*, causing Costello syndrome, in *FGFR3*, causing Achondroplasia, and other genes [33]. Since this clonal expansion develops with time, this mechanism is better compatible with the occurrence of mutation in men with advancing age but a selective advantage in the maternal germline cannot be excluded.

Another intriguing general issue regards individual genetic background which may affect the ability of DNA repair, thus allowing differential efficiency of repair and individual differences in the chance of developing mutation [28].

Concerning the specific role of *ACVR1*/Alk2 in gametogenesis, experiments in the mouse demonstrated that Alk2^−/−^ embryos cannot form Primordial Germ Cells (PGCs), and, in Alk2^+/−^ heterozygous embryos, the number of PGCs was reduced when compared with wild-type littermates, demonstrating that Alk2 is necessary for PGC formation in vivo [34]. We are not aware of any evidence of how an activating mutation might affect this process and other steps of the gametogenesis.

Although we cannot define the precise mechanism underlying the FOP recurrent mutation, the significant correlation between the methylation level and the germline mutation rate at CpG sites suggests that this mechanism may be causative of the FOP G>A substitution.

In our experience, one FOP patient who carried a disease causative mutation different from the recurrent c.617G>A substitution, c.774G>C leading to the R258S aminoacid change in the kinase domain of the protein, was also heterozygous for the c.44C>G substitution, causing the A15G aminoacid change in the protein signal peptide. The c.44C>G is reported in the dbSNP (rs13406336) with a global frequency of the minor allele of 0.007 according to 1000 Genomes. This variant was inherited from the healthy father and the cDNA analysis showed that it resulted in *cis* configuration with the R258S causative mutation, thus showing that it occurred in the paternal allele [35]. Sequencing of trios (father, mother, FOP affected patient) could detect analogous events that might demonstrate the parental origin of the causative mutation. In particular, for patients carrying the c.617G>A substitution, the occurrence in *cis* with the signal peptide variant would be useful to attribute the parental origin of the *de novo* recurrent mutation. However, this variant is rare, and we are not aware of any other FOP affected individual with such occurrence. Interestingly, a synonymous variant in exon 4, c.270G>A (dbSNP: rs2227861), has frequency of the G allele of 0.35 and A 0.65 according to 1000 Genomes, which makes it suitable for trios analysis.

## 3. Regulatory Genetic Variants in *cis*

DNA sequencing is the most important tool available to diagnose genetic diseases caused by single gene mutations and has uncovered a great number of causative variants that can be analyzed to establish genotype–phenotype correlations and disease mechanisms. In the case of rare monogenic disorders, sequencing of coding regions of protein encoding genes is mainly performed. Concerning FOP, sequencing is routinely carried out for diagnosis of FOP [36], which is required to confirm the clinical diagnosis.

In the previous section we describe the c.44C>G variant responsible for the A15G aminoacid change in the signal peptide of the *ACVR1* receptor protein, which is not a disease causing variant. Nevertheless, we cannot exclude that this rare variant in the signal peptide could affect the efficiency of transfer of the receptor protein to the plasma membrane; however, neither we nor others have done experiments to prove such a functional effect.

The study of the so called complex diseases, usually common conditions, has used other methods, such as Genome Wide Association Studies (GWAS), which have detected a huge amount of DNA variants, essentially single-nucleotide variants (SNVs), showing statistically significant association with complex disease phenotypic traits. The majority of these variants are common, having a frequency >1% in the population and, interestingly, are located outside of coding regions, likely involved in regulation of gene expression and, in few cases, proved to have a functional role [37]. Such a large mass of data required the creation of databases and analytical methods necessary to expand the initial essential information derived from the Human Genome Project. The Genotype-Tissue Expression (GTEx) project [38] is a public resource that provides important information on the regulation of gene expression in tissues also at the single gene level. GTEx has built a map of variants that play a role in gene regulation, named expression quantitative trait loci (eQTLs) [39]. In 2020, the GTEx Consortium published its final set of studies analyzing genotype data from almost 1000 post-mortem donors completely genotyped by whole genome sequencing and more than 17,000 RNA-seq samples across 54 tissue sites and 2 cell lines, reporting the detection of eQTLs in 48 tissues (GTEx Portal, see website). Although the aim of GTEx is mainly directed to the study of common diseases, the amount of information collected and publicly available is an invaluable resource for researchers who study human genetic diseases including rare genetic diseases caused by single-gene mutations. For these diseases, one important issue is how the concomitant occurrence of a pathogenic variant and a regulatory variant on the same allele may vary (increase or decrease) the expression of the mutated gene product, in particular when the pathogenic variant is present in heterozygosity. If such imbalance takes place, one can expect that the disease phenotype varies according to the amount of mutated protein. The variants in the GTEx portal can be viewed in dbSNP at NCBI (see website), the public domain archive for human single nucleotide variations, microsatellites, and small-scale insertions and deletions, that has a “genomic view” resource through which any single locus is placed in the human genome and can be further investigated by the UCSC genome browser. We then used the data of the ENCODE Consortium (see website) which has built a Registry of candidate *cis*-Regulatory Elements (CREs) based on signatures derived from DNase hypersensitivity, histone modifications (H3K4me3, H3K27ac), and CTCF ChIP-seq data. Currently, the Registry includes more than 900,000 CREs in the human genome classified either as distal enhancer-like or promoter-like or CTCF-only elements showed in different colors in the UCSF Genome Browser. Finally, we used the PROMO public resource (see website) to predict putative transcription factor binding sites (TFBS) in an unknown DNA sequence, which uses matrices constructed from TFBS defined in the TRANSFAC^®^ database.

We applied the above online repositories to study how variants in *cis* might affect the FOP disease manifestation.

In FOP, the basis for this study is made more favorable by the more than 95% frequency of patients carrying the same mutation in the *ACVR1* gene (see above); then, other variants in *cis* exert their regulatory effect, if any, on the expression of the allele that carries the same genetic mutation. Moreover, for FOP, a sort of indirect evidence for a dependence of phenotypic features on the allelic composition was provided in a mouse model described by Lees Shepard et al. [12]. Heterozygous mice carrying the Acvr1 R206H mutation in which the wild type allele was deleted showed great increase in injury-induced HO compared to the heterozygous ones. This finding suggests that the HO phenotype is dependent on a gene dosage effect.

**Figure 2:**
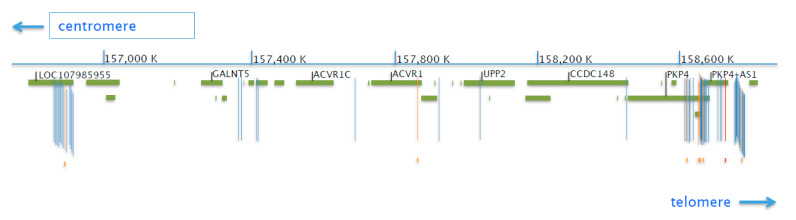
Map of the 62 eQTLs of the *ACVR1* gene (modified from the NCBI Sequence viewer). Green bars represent genes annotated in the NCBI sequence viewer at 30% magnification spanning a genomic region of around 2 Mb of the chromosome 2, from position 156,800,000 upstream of LOC107985955 to 158,816,000 in the *DAPL1* gene region. Vertical bars represent the position of each of the 62 single-nucleotide variants (SNVs) listed by the GTEx portal as affecting the *ACVR1* gene expression; colors are blue, orange for variants lying in a distal-like enhancer element, or red for the one lying in a promoter-like element. The small spots under the bars indicate the position of distal-like enhancer, orange color, or promoter elements, red color, according to ENCODE. Numbers above the map indicate the position in the chromosome according to the human genome GRCh38/hg38 release.

Accessing the GTEx portal, we obtained information on eQTLs that affect the expression of the ACVR1 gene, which consists of a list of 62 variants. These variants, ordered from the most centromeric to the most telomeric, are located in an interval of the Chromosome 2q spanning from position 156,878,023 to 158,771,340 that contains all the eQTLs listed in GTEx. The SNVs list in the GTEx portal reports, for each of them, the tissue where the effect on expression was detected. However, we know that *ACVR1* is expressed in a large variety of tissues and cell types; then, we may assume that an eQTL exerts its effect in many or most tissues. We then localized these variants using the dbSNP “genomic view” resource and the UCSC genome browser where CREs made available by the ENCODE Consortium (see website) are annotated. The map of the 62 eQTLs for the *ACVR1* gene in this interval is reported in Figure 2. It is interesting to note that only a few of the 62 variants map in close vicinity to the ACVR1 gene or inside the gene region, while most of them are located relatively far, many of them arranged in two large clusters. Another consideration was that eight of the 62 SNVs are located inside a CRE (7 defined as distal enhancer-like and 1 as promoter-like) as reported in Table 1.

It is likely that, because of linkage disequilibrium, most of the mapped variants are associated with one or a few true functional variants that we cannot identify but which could also be included among those present in the map, which affect the expression of the gene. Therefore, we chose some criteria to indicate the ones which we might consider as possible candidate functional variants. The main criteria applied here were the location inside a regulatory element (according to ENCODE), conservation among species, and change of transcription factor binding (either abolition or introduction) dependent on the change of nucleotide sequence in the alternative alleles. For each of them, we analyzed TFBS by the PROMO resource, comparing the sequence carrying the major allele flanked by 50 nucleotides on each side with the same sequence carrying the alternative allele; and we found that for three of them, the alternative variant does not affect the binding of transcription factors. The indication of a variant as a candidate suggests that it should be tested by appropriate experimental methods to verify its functional role.

A crucial point to evaluate the significance of a single variant in *cis* with the *ACVR1* causative mutation is the measure of allele-specific expression (ASE). This can be done through the analysis of RNA-seq reads that include heterozygous SNPs, allowing one to assign the read to an allele. Allelic imbalance occurs when the two alleles of a gene are differentially expressed and the magnitude of the imbalance can be quantified by allelic fold change (aFC) [40]. Although it is too simplistic to attribute an expression imbalance to a single variant, rather than to a cumulative effect of more variants on each allele, we cannot exclude that one or some of the variants within a haplotype have a stronger effect on ASE. Analysis of a specific allele requires the reconstruction of the phase of variants that are on the same chromosome, including the causative mutation. If a patient’s parents are available for genotyping, phase reconstruction can be attempted, or long read sequencing by new sequencing technology can be utilized to obtain allele phasing, and interpretation of available data sets by informatics means can be of great help.

Modifier genetic variants, of course, can be located in *trans*-acting genes, and we expect that many of them have a role in phenotype variability dependent on the individual genetic background, as described in the following section.

The knowledge of the complex pattern of regulatory variants which can cause up- or down-regulation of the mutant allele could provide an explanation on one of the determinants, likely more than one, of phenotypic variability. Specifically for FOP, variability of disease manifestation is intrinsic to the unpredictable and intermittent disease course, and is therefore complex to evaluate, but the data collected by the natural history studies [41] and those expected from the registry organized by the IFOPA [42,43] will potentially allow one to better categorize the disease phenotypic features, which in turn can be related to etiological factors, among them genomic data underlying regulation of gene expression.

In diseases in which a mutation causes a gain of function effect, like the *ACVR1* mutation in FOP, down-regulation of the mutant allele could potentially reduce the pathogenic consequence of the activating variants. Approaches to target allele-specific gene expression to treat genetic disorders by modifying a gain of function disease mechanism are reported for a number of conditions, such as Huntington disease and Facioscapulohumeral muscular dystrophy [44], and the advancements of genome editing technologies will certainly provide new strategies to treat genetic disorders by these means. On the other hand, considering the effect of the quantitative balance between wild type and mutated ACVR1, we can also hypothesize a strategy able to up-regulate the wild type allele to modify the wild type/mutant ratio, then the amount of mutant receptor available to the ligand compared to wild type. Examples of strategies to increase one gene copy by targeting endogenous regulatory elements, for instance, to rescue haplo insufficiency, are reported [44,45]. However, similar approaches to increase one gene copy aimed at changing the ratio between the two alleles, could be used to increase the amount of wild type Alk2 receptor. In the above cited article by Lees Shepard et al. [12], the authors propose a model in which wild-type *ACVR1* present in cells of heterozygous FOP individuals, although unable to transduce the signal, is able to bind activin A and functions as a competitive inhibitor for binding to the mutated ACVR1 receptor, thus limiting the activin-dependent osteogenic signaling through the mutated ACVR1. In this model, the wild-type/mutant ratio may modulate the amount of pathogenic signal; then, inducing an increase of wild-type versus mutant would increase the binding of ligand to wild-type and compete for binding to the mutated receptor.

## 4. A General Approach to Investigate Genetic Modifiers of FOP

We are well aware that the functional effect of variants acting in *cis* with the causative gene mutation is only a limited part of the phenotypic heterogeneity, since complex interactions between genes and between genes and other functional elements in the individual genome, commonly defined as “genetic background”, contribute to affecting the disease manifestation. This issue is frequently underestimated because most efforts are focused on the causative gene mutation and its functional effects, limiting the understanding of the connection between a particular disease and many functional pathways. In the case of FOP, we think it would be theoretically possible to carry out a genetic interaction analysis looking for variants in genes that have functional relationships with ACVR1 and the BMP signaling pathways, although such analysis would be made difficult by some characteristics of the disease, such as its episodic and unpredictable course, and by the multiple functional roles of BMPs in a wide variety of organs and tissues.

Animal models can be of help also to search for genetic modifiers, provided that it is possible to model the phenotypic variation and that it can be attributed to genetic variation. As an example, we cite a recently published article in which the authors aimed to identify genetic determinants that regulate the severity of the phenotype of craniosynostosis, a disease caused by activating heterozygous mutation in the *FGFR2* gene [46]. The purpose of this study was to identify potential modifiers of craniosynostosis phenotype in mice models with the disease causative mutation on two different congenic background strains. By comparing transcriptomes of cranial tissues from craniosynostosis mouse models of the two different strains, they describe potential modifiers affecting the disease severity.

Another example of this type of study is provided by the use of genetically diverse Drosophila strains, known as the Drosophila Genetic Reference Panel (DGRP), to identify components of the genetic background that affect symptoms and severity of the rare *NGLY1* deficiency [47]. As already mentioned, a Drosophila FOP model has been reported [4]; thus, in principle it would be possible to apply a similar approach to search for genetic modifiers also for the FOP phenotype.

One major limitation in the study of modifiers of FOP in humans is the lack of large families where different affected individuals with minimal difference in genetic background and showing differential phenotypic features could be analyzed. On the other hand, the great majority of FOP affected individuals carry the same *ACVR1* mutation; then, their genome can be interrogated in search for differences that could be associated to some phenotypic features. However, grouping FOP individuals in distinguishable categories on the basis of phenotype has not reached sufficient accomplishment up to now. Hopefully, the data collected by the FOP Registry will allow some reasonable grouping in the future and a method to analyze individuals from different groups could be designed. RNA-sequencing to analyze differentially expressed genes in individuals, carrying the R206H mutation, with different phenotypic features could be a useful approach, but the choice of cells from which RNA should be extracted and the time when this extraction should be performed might be a problem to avoid differences of gene expression caused by mechanisms not related to the effect of modifier genes. Cells collected from FOP patients at the time of diagnosis before any treatment is performed might be an initial approach. Fibroblasts from skin biopsies would be very useful, but, to use less invasive procedures, cells from hair bulb or from urine might be collected and treated to carry out an appropriate analysis of transcriptome to try to identify genes and pathways relevant for FOP pathophysiology related to phenotype diversity. Coupled to RNA-sequencing, whole genome sequencing of DNA extracted from the same cells could also provide information on variants associated with individuals in defined groups, possibly variants in non coding regulatory regions of genes involved in the wide spectrum of the functional pathways that sustain the FOP pathophysiology. The type of study we are conceiving here would require the availability of the above mentioned samples collected in an appropriate bio-bank, the use of appropriate computational methods to evaluate gene expression, variants in the genome, and significant interactions among gene functions and pathways. Therefore, presenting this sort of hypothetical program, we would foresee an interdisciplinary collaboration of the different types of researchers involved in the study of FOP, clinicians, geneticists, molecular biologists, immunologists, together with computational scientists.

## 5. Conclusions

In this work we aimed to discuss the issue of the mechanisms underlying the extremely high prevalence of the c.617G>A mutation in FOP patients and to trace genomic studies suitable for identifying variants which can modify some features of the disease phenotype. We think that the study of genetic modifiers in FOP is worthy to be undertaken in the FOP scientific community, not only to add more basic knowledge, but also to provide cues for translational approaches paving new avenues of investigation and innovative therapeutic strategies.

## 6. Methods

To study how variants in *cis* might affect the FOP disease manifestation, we retrieved data related to the tissue-specific expression of the *ACVR1* gene from the Genotype-Tissue Expression portal (GTEx). The data used for the analyses described in this manuscript were obtained from the GTEx Portal, then were combined with the dbSNP database at NCBI and the UCSC Genome Browser through which the available location of CREs from the ENCODE consortium can be visualized. Finally, we used the PROMO tool at the ALLGEN resource for the study of transcription factor binding sites in the DNA sequences containing the selected variants.

Cited Websites

- Genotype-Tissue Expression (GTEx)
  https://gtexportal.org/home/ (accessed on 4 February 2021)
- dbSNP at the NCBI
  https://www.ncbi.nlm.nih.gov/snp/ (accessed on 4 February 2021)
- ENCODE consortium
  https://www.genome.gov/Funded-Programs-Projects/ENCODE-Project-ENCyclopedia-Of-DNA-Elements (accessed on 4 February 2021)
- ENCODE at the UCSC Genome Browser
  https://genome.ucsc.edu/ENCODE/ (accessed on 4 February 2021)
- PROMO 3.0
  http://alggen.lsi.upc.edu (accessed on 4 February 2021)

## Figures and Tables

**Table 1:** List of the variants selected from the GTEx eQTLs affecting the expression of the *ACVR1* gene and located inside a candidate *cis*-Regulatory Region (CRE).

	dbSNP ID	Position	Alleles	Conservation	CRE	Effect on TF Binding
1	rs7586274	chr2:156896695 LOC107985955 intron	C/T 98/2	C: Homo, Chimp, Rhesus T: Mouse, Rat, Dog	EH38E2043429 distal enhancer-like	No effect
2	rs568810076	chr2:157866444 *ACVR1* intron 1	G repeat 7G/2G 97/35G/4G/3G	poor conservation	EH38E2043925 distal enhancer-like	Yes: alleles with 5G and 2G miss a WT1 bs compared to 7G
3	rs79473991	chr2:158621197 *PKP4* intron	A/G 98/2	A: Homo, Rhesus, Mouse, Dog	EH38E2044280 distal enhancer-like	No effect
4	rs59520356	chr2_158651353 *PKP4* intron	A/G 93/7	A: Homo, Rhesus, Mouse, Dog, Elephant	EH38E204429 distal enhancer-like	No effect
5	rs58540852	chr2:158658609 *PKP4* intron	A/T 93/7	A: Homo, Rhesus T: Mouse, Elephant	EH38E2044304 distal enhancer-like	Yes: the minor allele acquires a GATA1 bs and loses a STAT5A bs
6	rs73006932	chr2:158662549 *PKP4* intron	G/C 92/8	G: Homo, Rhesus, Mouse C: Dog, Elephant	EH38E2044306 distal enhancer-like	Yes: the minor allele loses ATF3, PR B, PR A bs
7	rs17231614	chr2:158724233 *PKP4*-AS1 intron	G/T 96/4	G: Homo, Rhesus, Mouse, Elephant	EH38E2044344 promoter-like	Yes: the minor allele loses ETF bs and acquires RXR-α bs
8	rs967380	chr2:158766477	T/C 20/80	T: Homo, Rhesus, Dog, Elephant	EH38E2044392 distal enhancer-like	No effect

dbSNP ID, variant accession number in the dbSNP database; Position reports the nucleotide position in chromosome 2q according to the human genome GRCh38/hg38 release; Alleles reports the frequency of the alternative alleles according to the 1000 Genomes Phase 3 dataset; Conservation reports conservation of the alleles across species as available in the UCSC Genome Browser; CRE reports the code of the *cis* Regulatory Element according to the ENCODE resource, annotated in the UCSC Genome Browser; the last column reports the effect on Transcription Factor binding sites analyzed by the PROMO resource at the ALGGEN server, comparing sequences carrying the alternative alleles. TF, Transcription Factor; bs, binding site.

## Data Availability

Not applicable.
